# Carbon Nanomaterials with SOD-like Activity: The Effect of the Ionic Strength

**DOI:** 10.3390/molecules29174098

**Published:** 2024-08-29

**Authors:** Andreia D. Veloso, Romeu A. Videira, Maria C. Oliveira

**Affiliations:** 1 Centro de Química-Vila Real (CQ-VR) and Chemistry Department, University of Trás-os-Montes e Alto Douro, 5001-801 Vila Real, Portugal; andreiav@utad.pt; 2 REQUIMTE/LAQV, Laboratory of Pharmacognosy, Chemistry Department, Faculty of Pharmacy, University of Porto, 4050-313 Porto, Portugal

**Keywords:** SOD, voltammetry, oxidoreductase, nanozyme, antioxidant, biomimetics, mechanism

## Abstract

Electrogenerated hydrophilic carbon (EHC) nanomaterials emerge as a highly attractive option for mimicking the activity of the superoxide dismutase enzyme (SOD) due to their exceptional water solubility and electron-transfer reversibility. Motivated by these properties, the EHC nanomaterials were utilized to assess the effect of ionic strength on the SOD-like activity. Superoxide anion radicals (O_2_^•−^) were generated using the hypoxanthine–xanthine oxidase system, with nitro blue tetrazolium chloride serving as the detecting system. A significant boost in the SOD-like activity was found via the addition of an electrolyte to the as-prepared nanomaterial solution. The effect of the electrolyte cation (Na^+^ and K^+^), as well as its counterion (Cl^−^, CH_3_COO^−^, and H_2_PO_4_^−^/HPO_4_^2−^) were analyzed. Based on these studies, a new formulation for the preparation of the carbon-based nanomaterial was established. It was demonstrated that the SOD-like activity follows an enzyme-type catalytic activity rather than the stoichiometric scavenging of the superoxide anion radical. It was concluded that 12.71 µg/mL of the EHC nanomaterial exhibits catalytic activity comparable to 15.46 µg/mL of the native Cu/Zn-SOD enzyme. This study provides a starting point for the development of a new nanotool to fight the oxidative stress associated with pathophysiological conditions where SOD activity is depleted.

## 1. Introduction

The superoxide dismutase enzymes (SODs) are a family of oxidoreductase enzymes found in all aerobic organisms that catalyze the dismutation of two superoxide anion free radicals (O_2_^•−^) into molecular oxygen (O_2_) and hydrogen peroxide (H_2_O_2_). They play a key role in maintaining cell redox balance and regulating reactive oxygen species (ROS)-dependent cell signaling pathways, while also providing protection against oxidative damage [1].

Driven by the promising therapeutic potential of SOD enzymes in combating oxidative stress-related diseases [2], extensive research has been dedicated to developing nanomaterials with SOD-like activity and the ability to overcome the limitations associated with the use of native enzymes in therapeutic applications [2,3,4,5,6]. The main limitation of SOD and native enzymes is their chemical stability. Enzymes are highly sensitive to environmental conditions, denaturing or losing activity under non-optimal temperature and pH conditions. They are typically single-use and require specific storage conditions. They also suffer from high production costs and complex preparation processes, making the scale-up for mass production challenging and economically unfeasible.

Numerous nanomaterials with activity akin to oxidoreductase enzymes have been developed, but less than 3% of these nanomaterials exhibit competence to mimic SOD activity. SOD-like properties have been found in carbon nanomaterials like fullerenes [7], hydrophilic carbon clusters [8], and graphene quantum dots [9]. However, the SOD-like activity exhibited by these nanomaterials is typically very low, highlighting the necessity of enhancing their intrinsic redox properties.

To modulate the redox properties of carbon-based nanomaterials, different approaches have been proposed. These include doping the nanomaterial with metals or heteroatoms [10,11,12], and functionalization with phenol-like groups [13] or with very specific molecules [14]. In the present study, a different strategy is proposed, based on the modulation of the ionic strength of the solution. The fine control of the ionic environment is expected to provide electrostatic guidance of the negatively charged O_2_^•−^ species to the nanomaterial active sites. This approach avoids the risk of toxicity often associated with the presence of metals, a relevant issue for nanomaterials’ application in the medical field.

To carry out this study, electrogenerated hydrophilic carbon nanomaterial, produced from graphite in a KH_2_PO_4_/K_2_HPO_4_ buffer (represented by the acronym EHC@phosphate) was used. Typically, as-prepared EHC@phosphate forms supramolecular entities which, in the presence of water molecules, turn into large discrete roundish agglomerates (up to 160 nm as estimated via AFM). These aggregates are assemblies of much smaller units (<15 nm), which are only evidenced when the electrolyte is removed. The disordered carbon network structures are dominated by a core of aromatic sp^2^ carbon atoms, functionalized with oxygenated groups such as quinones, epoxide, hydroxyl, and carboxyl [15,16]. This nanomaterial is also characterized by an anomalous high content of sodium ions (atomic ratio Na/C= 0.15–0.59).

EHC@phosphate shows promising potential for exhibiting SOD-like activity since it demonstrates electron-transfer reversibility [16], a prerequisite for the “ping-pong” mechanism played by SOD catalysts [17]. Additionally, it possesses outstanding water solubility and many other requirements for mimicking the activity of SODs, as will be described below.

We have previously shown that the removal of the electrolyte from the as-prepared nanomaterial has an important impact on the chemical reactivity and structure of the EHC@phosphate nanomaterial [15]. Hence, for a more comprehensive understanding of the catalytic activity of the EHC nanomaterial, no attempt was made to remove the electrolyte from the solution where it was electrogenerated.

Our work demonstrates that the new experimental approach can boost the SOD-like activity of the EHC nanomaterials to levels comparable to those of native enzymes. The ability of EHC nanomaterials to mimic the activity of SOD was evaluated spectrophotometrically using an indirect method based on the competition between *p*-nitro blue tetrazolium (NBT) and the nanomaterials for O_2_^•−^ generated via the hypoxanthine–xanthine oxidase (HX/XO) system.

## 2. Results and Discussion

**Figure 1:**
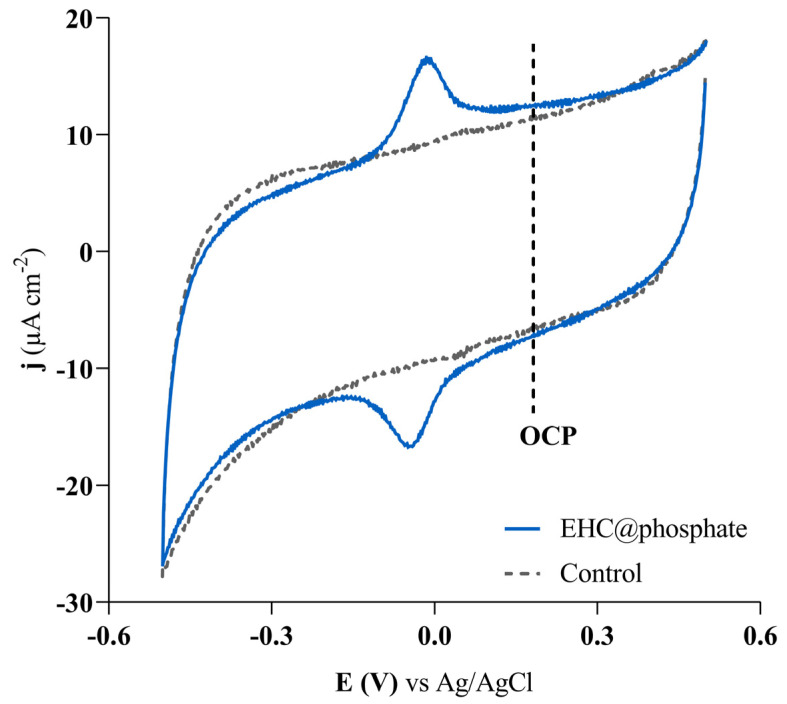
Cyclic voltammogram of the as-prepared EHC@phosphate solution (pH = 2.25) using a glassy carbon working electrode. It is also included the voltammogram recorded in the phosphate buffer solution, at the same pH as the nanomaterial solution (control), and a vertical dash line representing the open circuit potential (OCP). υ = 200 mV s^−1^.

The formal redox potential of as-prepared EHC@phosphate (pH = 2.25) was estimated via cyclic voltammetry within the range of −0.50 to +0.50 V vs. Ag/AgCl, Figure 1. It demonstrates a reversible couple with a formal potential of −0.020 V vs. Ag/AgCl, equivalent to 0.33 V vs. a Reversible Hydrogen Electrode (RHE), attributed to a two-electron transfer reaction involving quinone (Q)/hydroquinone (HQ) functionalities [16]. The cathodic peak was depicted as soon as the potential was scanned from the open circuit potential (OCP) towards more negative potentials, which demonstrates that the as-prepared nanomaterial is generated in the oxidized state. Although the redox potential value alone is not a definitive indicator of SOD-like activity in nanomaterials, it serves as a useful tool for evaluating their ability to catalyze the dismutation of superoxide anion free radicals. In fact, the redox potential of EHC@phosphate is very close to the intermediate value between the one-electron reduction potential of oxygen (−0.16 V vs. RHE) and the one-electron reduction potential of the superoxide anion radical (0.89 V vs. RHE), which is considered the ideal value for SOD activity in aqueous media [18]. The active center in native SOD enzymes exhibits a redox potential in the range of 0.2–0.45 V vs. RHE, further supporting the likelihood of SOD-like activity in EHC@phosphate.

**Figure 2:**
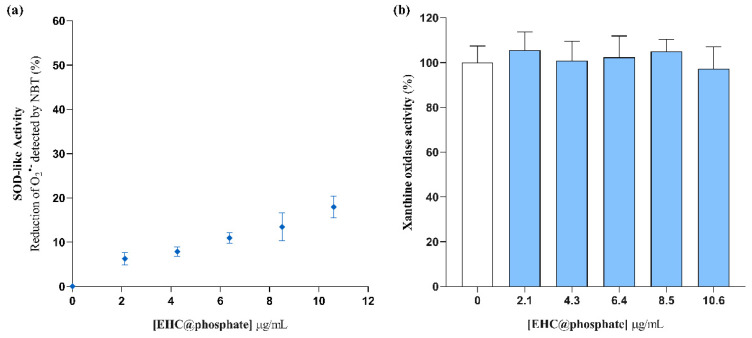
(**a**) SOD-like activity of EHC@phosphate, using the hypoxanthine–xanthine oxidase system to generate O_2_^•−^, and NBT for detection. (**b**) Effect of the EHC@phosphate nanomaterials on the XO activity. Statistical comparisons were made using one-way ANOVA followed by Dunnett’s multiple comparisons test. No statistical differences were detected.

The SOD-like activity of as-prepared EHC@phosphate was assessed at concentrations ranging from 0 to 11 μg/mL. Higher concentrations were not addressed due to the volume of solution required, which would compromise the feasibility of the analytical process, particularly the buffer capacity of the solution and the corresponding ionic strength (Table S1). Figure 2a shows that the SOD-like activity of EHC@phosphate increases progressively with increasing concentration of nanomaterial. To accurately calculate the SOD-like activity based on the decrease in the NBT reduction rate via O_2_^•−^, it is crucial to ensure that the nanomaterial does not produce false positives by either inhibiting the xanthine oxidase enzyme or directly reducing the NBT. Confirmation that the decrease in the rate of NBT reduction did not occur due to XO inhibition was obtained by following the kinetics of uric acid formation at 295 nm after replacing the NBT solution with an equal volume of buffer solution. The obtained results demonstrate that within the tested concentration range, EHC@phosphate does not alter the rate of O_2_^•−^ production (Figure 2b). Confirmation that NBT is not reduced by the carbon-based nanomaterial was ensured via a control assay conducted in the absence of the HX/XO system.

Although the data from Figure 2 provide evidence that EHC@phosphate reacts with superoxide anion free radicals, an apparent low activity is attained. Accordingly, it was not possible to determine the EC_50_—the concentration of EHC@phosphate required to reduce by half the rate of O_2_^•−^ detection via NBT.

**Figure 3:**
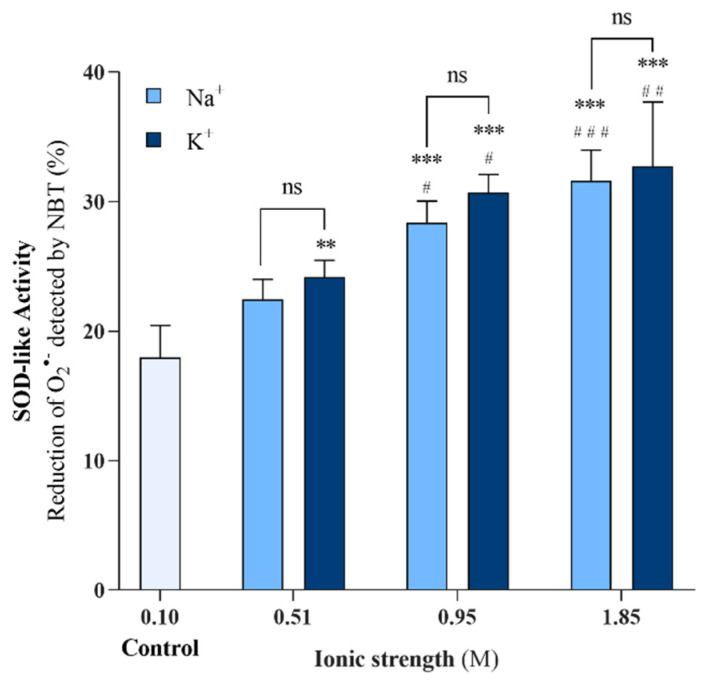
Effect of increasing ionic strength promoted by the addition of sodium acetate or potassium acetate on the SOD-like activity of EHC@phosphate (10.6 µg/mL). Statistical comparisons were made using two-way ANOVA followed by Sidak’s multiple comparisons test to compare groups with the same ionic strength and Tukey’s multiple comparisons test to compare each sample under study with the control and among themselves. ** and ***: significantly different from the control at *p* < 0.01 and *p* < 0.001. #, ##, and ###: significantly different from the corresponding 0.51 M at *p* ≤ 0.05, *p* < 0.01, and *p* < 0.001. n.s indicates no significant differences.

In our previous work, it was shown that electrolyte removal has a strong impact on EHC@phosphate’s structure and reactivity [15]. In this study, we explore the reverse effect, by investigating the influence of the concentration of electrically charged species in solution on the SOD-like activity of the carbon-based nanomaterial. The experiments were carried out under an experimental design that was able to evaluate the specific influence of different anions and cations, as seen in Figures S1 and S2. Firstly, the ionic strength of the as-prepared EHC@phosphate solution (ionic strength of 0.10 M) was raised by adding NaCH_3_COO or KCH_3_COO salts to achieve solutions with an ionic strength of 0.51, 0.95, and 1.85 M, while maintaining the same concentration of nanomaterial (10.6 µg/mL). These modified nanomaterial solutions were then utilized to evaluate the SOD-like activity of EHC@phosphate, as seen in Figure 3. The obtained results demonstrate a progressive and significant enhancement of the SOD-like activity of EHC@phosphate as the ionic strength increases, regardless of the cation used in the salt.

**Figure 4:**
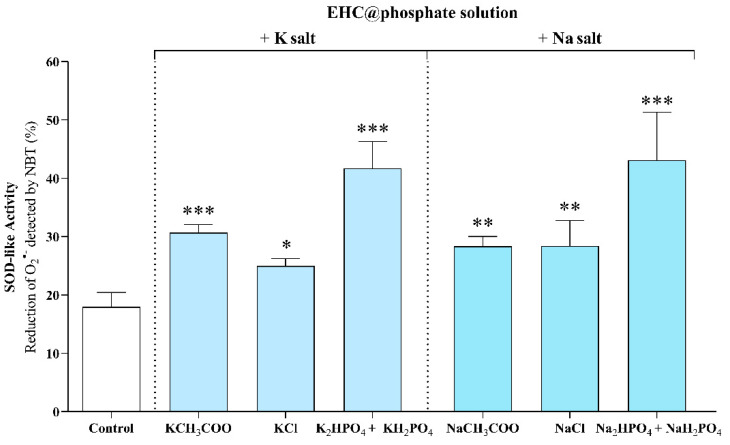
Effect of the counterion on the SOD-like activity of EHC@phosphate by increasing the ionic strength through the addition of different salts. The salts were added maintaining constant the total cation concentration (0.93 M) and the nanomaterial concentration (10.6 µg/mL). Statistical comparisons were made using one-way ANOVA followed by Holm–Sidak’s multiple comparisons test. *, **, and ***: significantly different from the control at *p* ≤ 0.05, *p* < 0.01, and *p* < 0.001, respectively.

Secondly, a set of experiments was conducted using the same salt cation (sodium or potassium) with different anions (Cl^−^, CH_3_COO^−^, and H_2_PO_4_^−^/HPO_4_^2−^) to increase the ionic strength of the as-prepared EHC@phosphate solution, and the effect on the SOD-like activity was analyzed. The salts were added while maintaining the total cation concentration constant (0.93 M) as well as the nanomaterial concentration (10.6 µg/mL). As shown in Figure 4, the K_2_HPO_4_/KH_2_PO_4_ or Na_2_HPO_4_/NaH_2_PO_4_ salts induce a greater increase in EHC@phosphate activity than their equivalent salts with chloride or acetate anions. It can be concluded that the cations Na^+^ and K^+^, along with the phosphate species HPO_4_^2−^ and H_2_PO_4_^−^, likely promote the reorganization of EHC@phosphate. The new organization provides electrostatic guidance for the approach of the superoxide anion radical to the catalytic sites (quinone/hydroquinone groups), resulting in greater efficiency in reacting with superoxide anion radicals.

**Figure 5:**
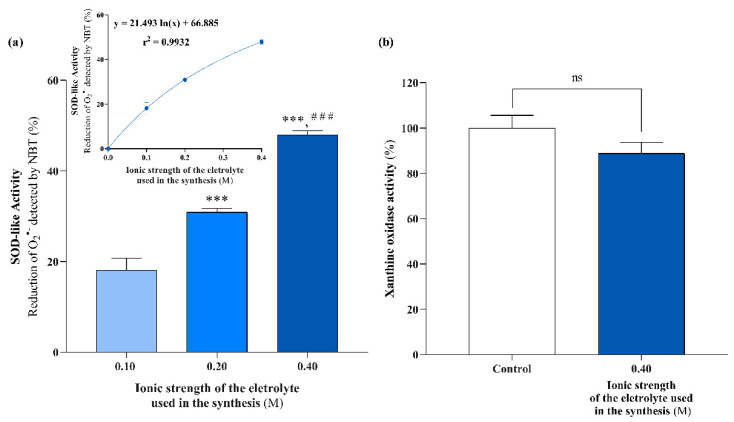
(**a**). Evaluation of the effect of the electrolyte’s ionic strength on the SOD-like activity of EHC generated in potassium phosphate buffer solutions with increasing ionic strength (SOD-like activity was assessed for a final concentration of nanomaterials of 10.6 μg/mL). Results are presented as mean ± standard deviation from at least 2 independent assays. Statistical comparisons were made using one-way ANOVA followed by Tukey’s multiple comparisons test. *** Significantly different from the sample synthesized in the electrolyte with I = 0.10, and ### represents significantly different from the sample synthesized in the electrolyte with I = 0.20 at *p* < 0.001, respectively. (**b**) Effect on XO activity of 10.6 μg/mL of EHC@phosphate synthesized in the electrolyte with higher ionic strength (I = 0.40 M) (**b**). Results are presented as mean ± standard deviation from 3 independent assays. Statistical comparisons were made using an unpaired two-tailed *t*-test. n.s indicates no significant differences.

Previous studies have shown that the ionic strength of the electrolyte used during the synthesis of EHC@phosphate does not affect the concentration of the generated nanomaterial [14]. Therefore, we also evaluated the SOD-like activity of EHC@phosphate samples synthesized in phosphate buffer solutions (KH_2_PO_4_ + K_2_HPO_4_) with ionic strengths of 0.1, 0.2, and 0.4 M to eliminate the need for post-synthesis adjustment of the electrolyte concentration. The nanomaterials synthesized in these electrolyte solutions were tested at a concentration of 10.6 µg/mL. As shown in Figure 5a, the obtained results confirm that the ionic strength of the synthesis solution significantly influences the capacity of EHC@phosphate to react with O_2_^•−^, with the dependence of SOD-like activity on the ionic strength following a trend that can be described with a logarithmic function (inset). Additionally, it was confirmed that the increase in SOD-like activity does not result from inhibition of the XO enzyme (Figure 5b).

**Figure 6:**
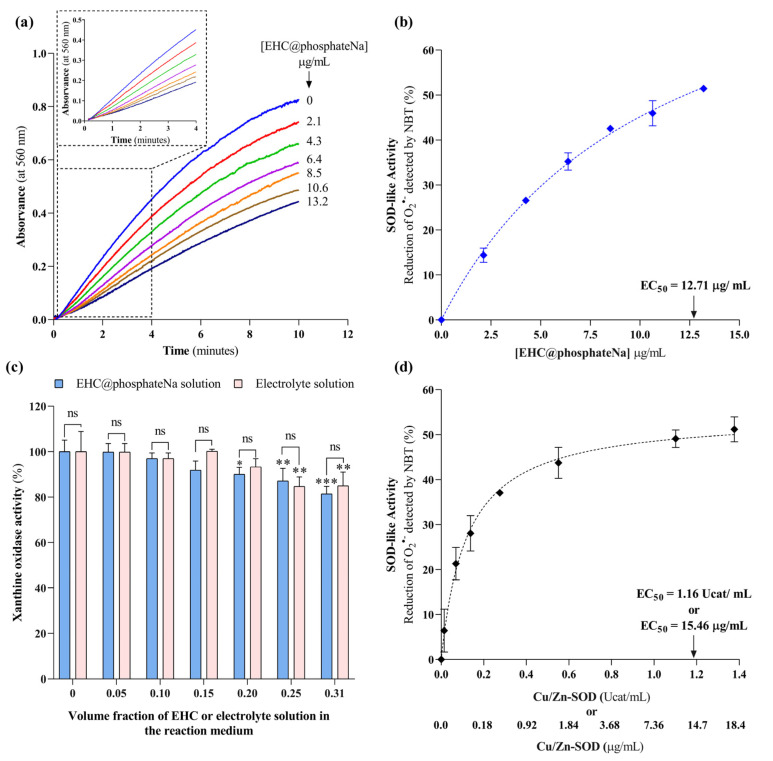
Evaluation of the SOD-like activity of EHC@phosphate synthesized in sodium phosphate buffer solution with I = 0.40 M. (**a**) Reaction time curves of the colorimetric reaction of NBT with O_2_^•−^ in the absence and presence of increasing concentrations of EHC nanomaterials. Inset: enlarged initial linear portion of the reaction time curve. (**b**) Effect of increasing EHC@phosphateNa concentration on the reduction of O_2_^•−^ detected via NBT. (**c**) Effect of the volume fraction of EHC@phosphateNa or its respective electrolyte solution on XO activity. Each volume fraction corresponds to the respective EHC concentration presented in (**a**). Results are presented as a percentage of the control (XO activity in the absence of nanomaterials/electrolyte). (**d**) Effect of increasing the concentration of Cu/Zn-SOD on the rate of O_2_^•−^ reduction detected via NBT, expressed as micrograms per milliliter (µg/mL) and catalytic units per milliliter (Ucat/mL), on the rate of O_2_^•−^ reduction detected via NBT. Statistical comparisons were made using two-way ANOVA followed by Dunnett’s multiple comparisons test to compare the effect of different concentrations with the control and Sidak’s multiple comparisons test to compare the effect of the sample with the effect of the electrolyte. *, **, and *** represents significantly different from the control at *p* ≤ 0.05, *p* < 0.01, and *p* < 0.001, respectively. n.s. indicates no significant differences.

To envisage applications in nanomedicine, it is preferable to regulate the ionic strength of carbon nanomaterials solutions with sodium salts rather than potassium salts, due to the potential adverse effects of elevated extracellular potassium levels on human cell function, especially in brain and muscle tissues [19]. Thus, we also synthesized EHC@phosphate using a sodium phosphate electrolyte (Na_2_HPO_4_ + NaH_2_PO_4_, 0.2 M) with an ionic strength of 0.4 M. The new formulation, designated as EHC@phosphateNa, was used to evaluate its SOD-like activity across concentrations ranging from 0 to 13.2 µg/mL. Figure 6a shows the time-dependent curve of absorbance at 560 nm as the nanomaterial concentration increases and the range where the kinetics of NBT reduction by O_2_^•−^ follows a linear relationship. EHC@phosphateNa demonstrates a SOD-like activity dependence on the nanomaterial concentration (Figure 6b) that resembles the saturation curve observed with the native SOD enzyme (Figure 6d). The data in Figure 6b were normalized against paired control assays conducted without the nanomaterial but containing equivalent volumes of electrolyte solution, to mitigate any potential inhibitory effects on the XO enzyme. Hence, the SOD-like activity depicted in Figure 6b accurately reflects the activity of EHC@phosphateNa, allowing for the determination of an EC_50_ value of 12.71 µg/mL.

As depicted in Figure 6c, concentrations of nanomaterial exceeding 8.5 μg/mL (equivalent to a volume fraction of 0.2 in the reaction medium) result in a decrease in XO activity. Specifically, reductions of 12.9% and 18.6% in XO activity are detected for EHC@phosphateNa concentrations of 10.6 and 13.2 μg/mL, respectively. To investigate the cause of this, we examined whether the reduction in XO activity results from the direct effects of EHC@phosphateNa on the enzyme or from the concomitant increase in electrolyte concentration. The data presented in Figure 6c suggest that the reduction in XO activity is primarily linked to the electrolyte rather than the nanomaterial (e.g., fluctuations in the pH). Therefore, EHC@phosphateNa does not inhibit the XO enzyme in the concentration range where it exhibits high SOD-like activity.

One limitation of the NBT assay in assessing SOD-like activity is its inability to distinguish between the catalytic dismutation and the stoichiometric scavenging of the superoxide anion radical via the nanomaterial. However, a detailed examination of the data provides some insight into this issue. Under control assays, the rate of O_2_^•−^ production and consumption is 21.30 nmol/min/mL and 23.02 nmol/min/mL, respectively, assessed via the stoichiometry of the uric acid production and the NBT reduction. Note that NBT reduction is performed under conditions that are not limited by the NBT concentration, which is 800 µM (equivalent to 597.3 µg/mL), a significantly higher concentration than the concentration of nanomaterials (0–13.2 µg/mL). The NBT concentration also exceeds that required to detect all O_2_^•−^ generated during the assay. Hence, the SOD-like activity dependence on the nanomaterial concentration follows a profile, characterized by a saturation curve that approaches a 50% O_2_^•−^ reduction rate. Consequently, for nanomaterial concentrations higher than 12.71 µg/mL, the rate of O_2_^•−^ generated by the hypoxanthine–xanthine oxidase becomes the limiting factor of the SOD-like activity exhibited by the nanomaterial. This behavior is consistent with an enzyme-type catalytic activity rather than with a stoichiometric scavenging activity [20]. Accordingly, a concentration-dependent saturation curve is observed with the native Cu/Zn-SOD enzyme (Figure 6d), with an EC_50_ value of 15.46 µg/mL, representing the concentration needed to reduce the superoxide radical anion detected via NBT by half. This allows for the calculation of a catalytic constant of 9.99 × 10^7^ M^−1^ s^−1^ for Cu/Zn-SOD, a value consistent with that reported in the literature for this enzyme [21]. Even though the unknown molecular mass of the nanomaterial prevents the calculation of the catalytic constant, these data allow us to infer that 12.71 µg/mL of NHC@phosphateNa exhibits a catalytic activity comparable to 15.46 µg/mL of the native Cu/Zn-SOD enzyme. It is also important to highlight that, whereas the native SOD enzyme features only a single catalytic site, a single nanoparticle of EHC@phosphate can host multiple redox-active functional groups with potential catalytic activity. This includes quinone and hydroquinone groups, as evidenced with the voltammetric data, located on the surface of a structure predominantly made up of sp^2^ carbon atoms [15].

**Figure 7:**
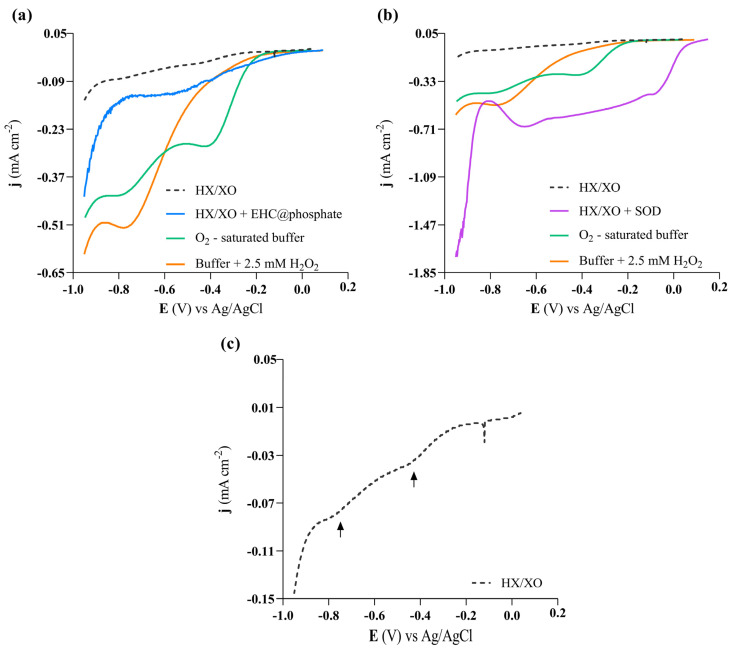
Linear voltammograms on Au electrode in solutions containing phosphate buffer and superoxide radical anion (generated in 8 μg/mL of xanthine oxidase + 0.20 mM of hypoxanthine, XO/HX) and EHC@phosphate solution (26.38 μg/mL; pH = 7.0) (**a**) or native SOD enzyme (36 μg/mL) (**b**). The voltammogram of the phosphate buffer is enlarged in (**c**). (**a**,**b**) also include blank assays (just containing HO/HX or O_2_ saturated phosphate buffer or phosphate buffer with H_2_O_2_). The arrows in (**c**) highlight the voltammetric peaks corresponding to the self-dismutation of the superoxide radical anion. υ = 50 mV s^−1^.

To mimic the activity of native SOD enzymes, it is imperative that the EHC@phosphate generate molecular oxygen and hydrogen peroxide from the reaction with two superoxide anion radicals at a rate higher than the self-dismutation rate of the superoxide anion free radical. Since molecular oxygen and hydrogen peroxide are both electroactive, the formation of these molecules was followed in the absence and presence of EHC@phosphate (26.38 μg/mL) via linear voltammetry in a solution containing O_2_^•−^ generated in situ via the HX/XO system, as shown in Figure 7a. For comparative purposes, a positive control was also performed by replacing the nanomaterial with the native SOD enzyme (36 µg/mL), as seen in Figure 7b. To identify the peaks corresponding to the formation of O_2_ and H_2_O_2_, voltammograms of blank solutions (0.1 M phosphate buffer solution, pH 7.40) saturated with O_2_ or supplemented with 2.5 mM H_2_O_2_ were also recorded. The spontaneous decomposition of the superoxide radical anion is also individualized in Figure 7c to highlight its low contribution in the presence of EHC@phosphate.

The oxygen reduction reaction (ORR) on the gold working electrode is characterized by an onset potential at approximately −0.20 V and two peaks at −0.39 V and −0.78 V. The first peak corresponds to the 2e^−^ reduction of O_2_ to H_2_O_2_, whereas the second peak is ascribed to the 2e^−^ reduction of H_2_O_2_ to H_2_O. In a phosphate buffer solution containing solely H_2_O_2_, only the second peak is detected (Figure 7a). Interestingly, the dismutation of the superoxide anion radicals catalyzed by the native SOD enzyme results in a broad band in the voltammogram, merging two peaks (Figure 7b). In comparison to the voltammogram of the O_2_ saturated solution, both the onset potential and the first peak potential are shifted by approximately 200 mV towards more positive potentials, whereas the second peak is shifted by approximately 130 mV. These findings suggest that the products generated by the SOD enzyme are much more electroactive than free O_2_ and free H_2_O_2_. It is probable that these products are adducts formed through strong intermolecular forces, such as hydrogen bonds and van der Waals interactions, between the enzyme and O_2_ and H_2_O_2_ molecules. The voltammogram obtained in the presence of EHC@phosphate also shows a significant broad band in the range of potentials observed with the native SOD enzyme, although with a reduced current density (Figure 7a). This behavior contrasts with the spontaneous self-dismutation of the superoxide anion radical, which is characterized by a voltammogram that only shows peaks corresponding to free O_2_ and free H_2_O_2_ (Figure 7c). Additionally, the current magnitude related to the self-dismutation of the superoxide anion radical is significantly lower compared to that observed in the presence of carbon-based nanomaterials (Figure 7a). Therefore, the voltammetry results also support the idea that EHC@phosphate nanomaterials promote the dismutation of superoxide anion radicals through a mechanism resembling that of the native SOD enzyme.

To fully understand the EHC catalytic behavior, a comprehensive comparative characterization of the nanomaterials prepared in both low and high concentrations of phosphate buffer (e.g., I = 0.10 and 0.40 M) would be necessary. However, this analysis falls outside the scope of this paper, requiring a separate study. Even though, given the similarity of the voltammetric response of the EHC solutions prepared in low and high concentrations of phosphate buffer) [16], we hypothesize that the same catalytic sites (quinone/hydroquinone groups) are displayed, regardless of the ionic concentration of the system. In this way, the electrolyte ions may promote the rearrangement of the carbon network layers within the hydrated clusters, enhancing the interlayer allocation of the superoxide ions, and, consequently, their interaction with the catalytic sites.

## 3. Materials and Methods

### 3.1. Experimental Design Overview and Material Specifications

This study was conducted using an experimental design with four main steps. In the first stage, the synthesis of the nanomaterials was conducted in a potassium phosphate buffer solution prepared with anhydrous potassium dihydrogen phosphate (KH_2_PO_4_, 99.995%, Merck, Darmstadt, Germany) and anhydrous dipotassium hydrogen phosphate (K_2_HPO_4_, 99.99%, Merck) with a total ionic strength of 0.1 M. It is important to emphasize that the synthesis process resulted in a significant reduction in the K^+^ concentration in the anodic compartment (assessed via atomic emission spectroscopy) to charge balance the substantial increase in H^+^. Consequently, after synthesis, the pH of the nanomaterial solution was adjusted to 6.1 (the minimum pH within the effective buffer capacity range), using 4 M KOH (98%, Panreac, Barcelona, Spain) to simultaneously restore K^+^ levels. This adjustment caused minimal change in solution volume (less than 0.9%). This solution was then used to evaluate SOD-like activity and for subsequent modulation of ionic strength prior to SOD-like activity assessment.

To the EHC solution (after pH adjustment), NaCH_3_COO (>99%, labkem, Porto, Portugal), KCH_3_COO (>99%, labkem), NaCl (99.5%, Merck), KCl (99.5–100.5%, Sigma-Aldrich, St. Louis, MO, USA), Na_2_HPO_4_ (99–101%, labkem), NaH_2_PO_4_ (>99%, labkem), K_2_HPO_4_ (99.99%, Merck), or KH_2_PO_4_ (99.995%, Merck) were subsequently added as solids to evaluate the effect of the cation and anion on the SOD-like activity. In a third stage, the nanomaterials were synthesized in potassium phosphate buffer solutions prepared with different ionic strengths (I = 0.1, 0.2, and 0.4 M), adjusted with the K_2_HPO_4_ (99.995%, Merck) and KH_2_PO_4_ (99.995%, Merck) salts. Whenever necessary, the pH was adjusted before the SOD analysis.

At the last stage, the nanomaterials were synthesized in a sodium phosphate buffer keeping the total ionic strength fixed at 0.4 M (NaH_2_PO_4_, >99%, labkem and Na_2_HPO_4_·2H_2_O, 99–101%, labkem) and the SOD-like activity was evaluated.

For the SOD-like activity, nitro blue tetrazolium chloride (NBT, C_40_H_30_Cl_2_N_10_O_6_, 98+%, Alfa Aesar, Heysham, Lancashire, UK) was used, as well as xanthine oxidase from bovine milk (XO, Sigma-Aldrich), hypoxanthine (HX, C_5_H_4_N_4_O, >99%, Alfa Aesar), EDTA disodium salt dihydrate (Na_2_C_10_H_14_N_2_O_8_.2H_2_O, 99.0–101.0%, Panreac), and KH_2_PO_4_ (99%, Fisher Chemicals, Pittsburgh, PA, USA). All reagents were used without additional purification, and all the solutions were prepared using ultrapure water (resistivity ≥ 18 MΩ·cm, Millipore Water System). A schematic representation of the experimental design is available in the Supplementary Materials (Figures S1 and S2), detailing the pH, ionic strength, and concentrations of all components in each experiment. The ionic strength was calculated using the following formula:

$$I=\frac{1}{2}\sum_{i=1}^{n}c_{i}z_{i}^{2}$$

where *c_i_* is the concentration of the ion in the solution, expressed in mol/L (molarity); *z*_*i*_ is the charge of ion *i*; and *n* is the total number of different types of ions present in the solution.

### 3.2. Synthesis of Electrogenerated Hydrophilic Carbon (EHC) Nanomaterials

EHC nanomaterials were synthesized via an electrochemical approach that was previously described [15,16]. Briefly, the electrolysis was performed under galvanostatic polarization for one hour (I = 60 mA). A three-compartment cell with graphite rods (99.997%, Goodfellow, Cambridge, UK) and a saturated calomel electrode were used. A phosphate buffer was used as the electrolyte (0.025 M K_2_HPO_4_ + 0.025 M KHPO_4_, pH 6.90). After the galvanostatic assay, the solution from the anodic compartment was collected and stored in dark glass vials at −18 °C. The concentration of EHC nanomaterials was determined via TOC analysis (Total Organic Carbon), resulting in a concentration of 17.6 ± 2.6 mg C/L (n = 5). The carbon content was converted into mass concentration based on the elemental composition of the nanomaterial obtained via XPS [15], resulting in a concentration of 42.53 ± 0.84 µg/mL.

### 3.3. SOD-like Activity Assay

The EHC nanomaterial (0–10.6 μg/mL) was added to phosphate buffer solution (100 mM KH_2_PO_4_, 5 mM EDTA, pH 7.40) supplemented with 800 µM NBT and 8 µg protein/mL xanthine oxidase (specific activity = 0.7 U/mg of protein). The mixture was incubated for 5 min at 37 °C under constant stirring. The reaction was started via the addition of 200 µM hypoxanthine, and the kinetics of NBT reduction via O_2_^•−^ was followed for 10 min by recording the absorbance at 560 nm in a UV-Vis-Varian Cary 50 spectrophotometer (Agilent Technologies, Santa Clara, CA, USA) equipped with a cuvette thermostat block and an automatic magnetic stirring system. As shown in Figure S3, the EHC nanomaterial does not absorb in the visible range. Under the control condition assays (i.e., absence of EHC), the concentration of reduced NBT increases at a constant rate of 9.208 µM/min (Δε_NBT_reduced__ = 12.800 mM^−1^·cm^−1^) for at least the first 4 min. The decrease in the NBT reduction rate, which reflects the nanomaterial’s ability to scavenge the O_2_^•−^, is used as a quantitative indicator of its SOD-like activity as previously described for biological samples [22]. For each EHC concentration tested, a paired control test was also carried out, in which the nanomaterial solution was replaced by an equal volume of electrolyte solution with the same composition, pH, and ionic strength. Positive control assays were performed in the absence of nanomaterial and the presence of increasing concentrations of SOD native enzyme. Unless otherwise stated, all results are presented as mean ± standard deviation from 3 independent assays. Detailed information on the volumes and concentration of each solution used in both EHC and control assays is available in Tables S1 and S2.

When the O_2_^•−^ is generated via 1 U of xanthine oxidase, one catalytic unit of SOD-like activity is defined by the amount of nanomaterial required to decrease the slope of NBT reduction by 50%. The EC_50_ obtained for the SOD enzyme was used to determine its catalytic constant (*K_cat_*). *K_cat_*, also known as the turnover number, is the maximum number of substrate molecules an enzyme can convert per unit of time when completely saturated with a substrate. For this purpose, the following formula was used: $K_{cat}=\frac{K_{NBT}\times [NBT]}{{EC}_{50}}$, where *K_NBT_* = 5.94 × 10^4^ M^−1^ s^−1^ [23].

### 3.4. Xanthine Oxidase Activity Assay

To accurately assess the SOD-like activity of nanomaterials, it is crucial to ensure that both the nanomaterial and its electrolyte do not interfere with the system used to generate the superoxide anion free radicals. Thus, the effects of increasing concentrations of EHC (0–10.6 μg/mL) and its electrolyte solution on xanthine oxidase activity were also evaluated. This was assessed by monitoring the kinetics of uric acid formation at 295 nm over a 10 min timeframe. The assays were conducted using the same setup as described for the evaluation of SOD-like activity, with NTB replaced by an equivalent volume of phosphate buffer in the reaction medium. The catalytic activity of XO was determined via the uric acid production rate (Δε_uric acid_ = 11,000 mM^−1^·cm^−1^), and the effects of nanomaterial and its electrolyte are expressed as % of control and are presented as mean ± standard deviation from 3 independent assays.

The specific activity of the xanthine oxidase was calculated using the following formula:

$$Specific\;activity=\frac{Total\;enzyme\;activity(U/mL)}{Total\;protein\;amount(mg/mL)}$$

For the XO enzyme, the enzyme activity was 0.7 U/mg protein. In the reaction medium, 8 µg protein/mL XO was added corresponding to 0.0056 U/m using hypoxanthine as the substrate.

### 3.5. Voltammetry

The electrochemical characterization of the as-prepared EHC@phosphate solution was carried out at room temperature in a single-compartment cell containing three electrodes: a carbon glass working electrode (GCE from ALS Co. (Tokyo, Japan) with a diameter of 6 mm), an Ag/AgCl (NaCl 3 M) (ALS Co.) reference electrode, and a platinum counter electrode. Before each assay, the working electrode surface was polished with an alumina slurry (0.3 µm), rinsed with ultrapure water, and ultrasonicated in water. The EHC solution was purged with argon for 30 min, and an argon atmosphere was maintained throughout the cyclic voltammetric assays.

To characterize the species resulting from the reaction of the carbon nanomaterial or native SOD (Cu/Zn superoxide dismutase from bovine erythrocytes, Sigma-Aldrich) with the superoxide ion species, the same electrochemical set-up but with a working gold electrode (ALS Co., ϕ= 2 mm) was used. The experiments were carried out at 37 °C in a 23.68 μg/mL EHC solution in which the pH was adjusted to pH 7.00. Before the voltammetric characterization, 8 μg/mL of xanthine oxidase and 0.20 mM of hypoxanthine were added to guarantee the formation of the superoxide ion.

## 4. Conclusions

The voltammetric behavior of the electroactive functional groups present in the EHC nanomaterial (quinone/hydroquinone), including their formal redox potential and chemical reversibility, proved to be reliable indicators of the EHC’s ability to mimic native SOD enzymes. We have demonstrated that the SOD-like activity of EHC nanomaterials can be boosted by increasing the ionic strength of either the synthesis solution or the EHC solution after synthesis. It was also concluded that the SOD-like activity of the EHC nanomaterial is driven by a catalytic dismutation reaction rather than by the stoichiometric scavenging of the superoxide anion radicals. Na_2_HPO_4_/NaH_2_PO_4_ electrolytes were found to induce a greater increase in EHC activity than their equivalent salts with chloride or acetate anions. Accordingly, 12.71 µg/mL of the EHC nanomaterial generated in a 0.2 M Na_2_HPO_4_/NaH_2_PO_4_ solution exhibit a catalytic activity comparable to 15.46 µg/mL of the native Cu/Zn-SOD enzyme.

Due to the EHC nanomaterial’s high ability to mimic SOD activity, it holds significant promise for therapeutic applications aimed at preventing or alleviating diseases associated with excess O_2_^•−^ and/or SOD deficiency.

## Data Availability

Data are contained within the article and Supplementary Information.

## Supplementary Materials

The following supporting information can be downloaded at: https://www.mdpi.com/article/10.3390/molecules29174098/s1, Figure S1: Overview of the experimental design, divided into four steps; Figure S2: Outline of steps 1 to 4 in the experimental procedure; Figure S3: Spectra of EHC, NBT and EHC+NBT solutions; Table S1: Conditions used for the SOD-like activity of EHC nanomaterials assay; Table S2: Conditions used for the SOD activity assay.
