# Supplementary material for: Carbon Nanomaterials with SOD-like Activity: The Effect of the Ionic Strength
Source: Molecules. 2024 Aug 29;29(17):4098. doi: 10.3390/molecules29174098 (PMC11397398; doi:10.3390/molecules29174098)
Supplement: Supplementary file 1 [file molecules-29-04098-s001.zip › molecules-3114029-supplementary.pdf]

Supporting Information

Carbon nanomaterials with SOD-like activity: the effect of the ionic strength

Andreia D. Veloso[1], Romeu A. Videira[2*] and Maria C. Oliveira[1*]

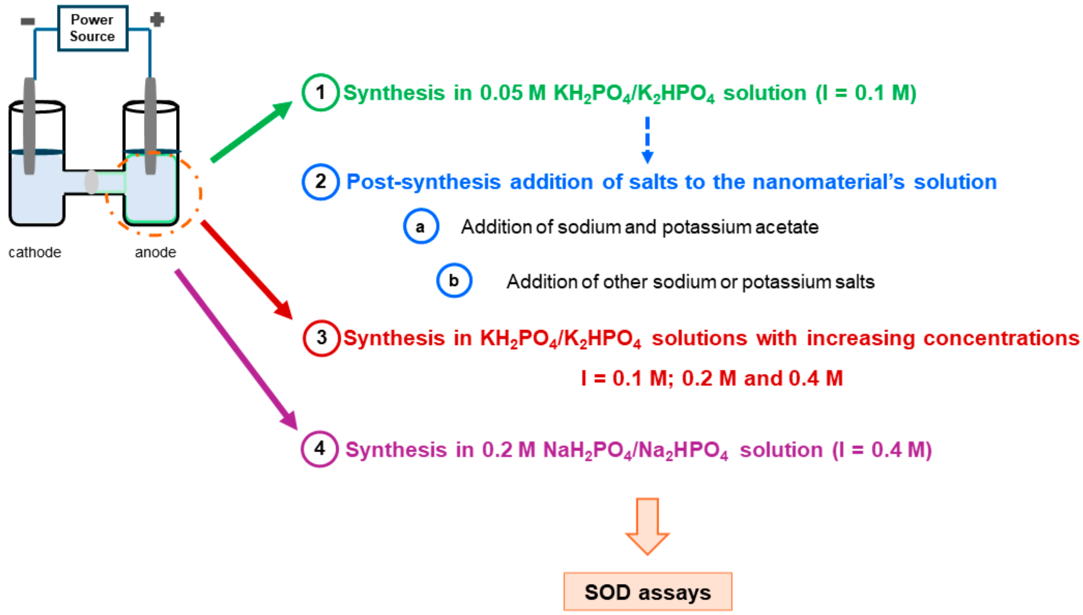

Figure S1. Overview of the experimental design, divided into four steps.

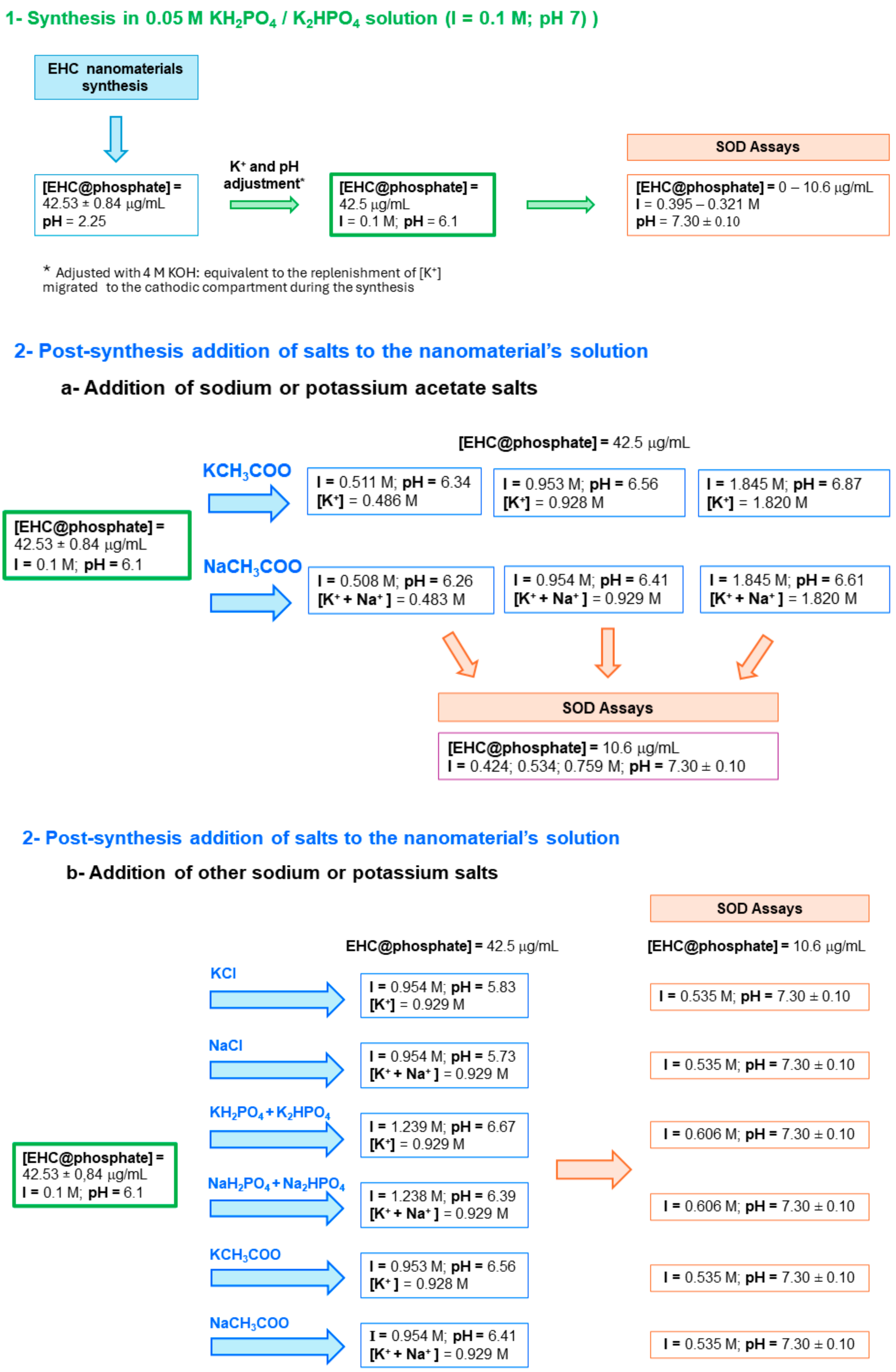

Figure S2. Outline of steps 1 to 4 in the experimental procedure.

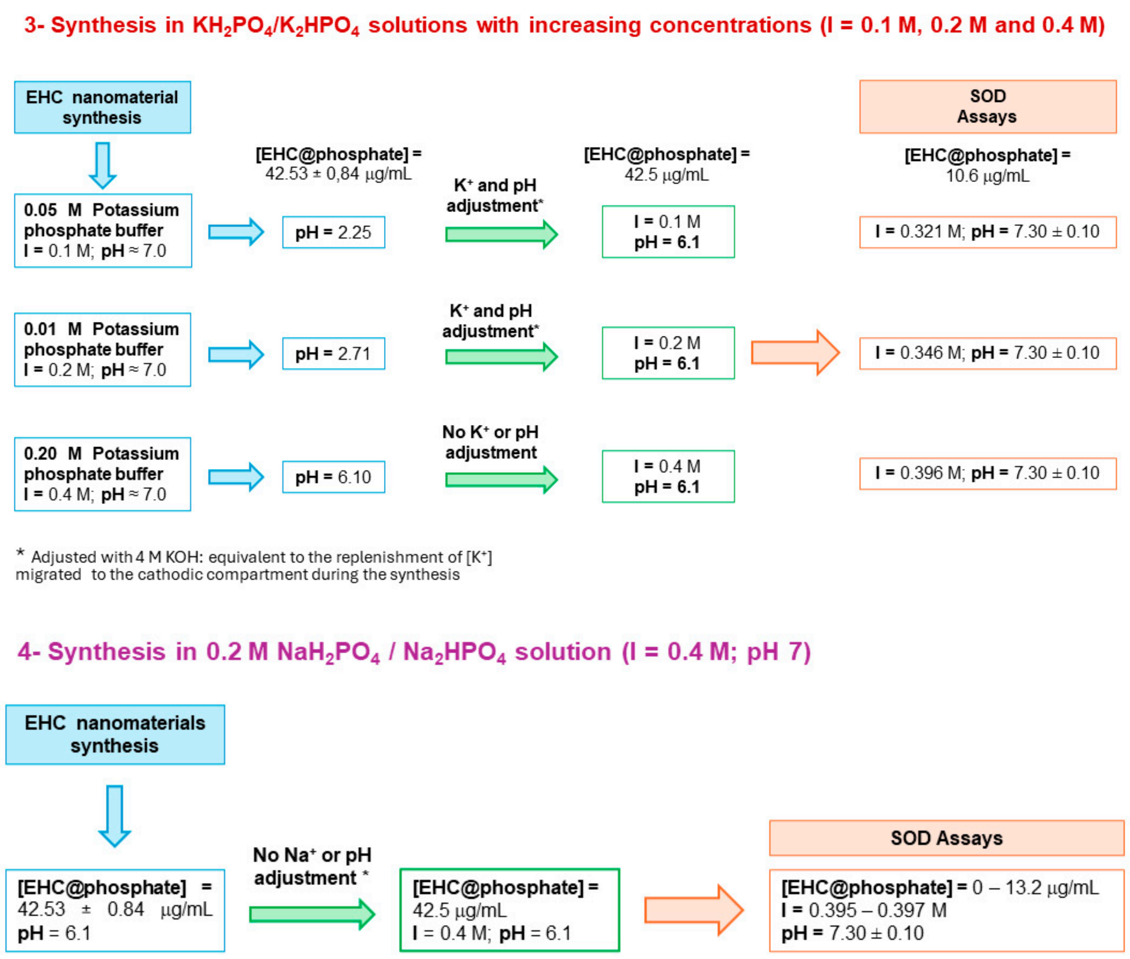

Figure S2. Outline of steps 1 to 4 in the experimental procedure (cont.).

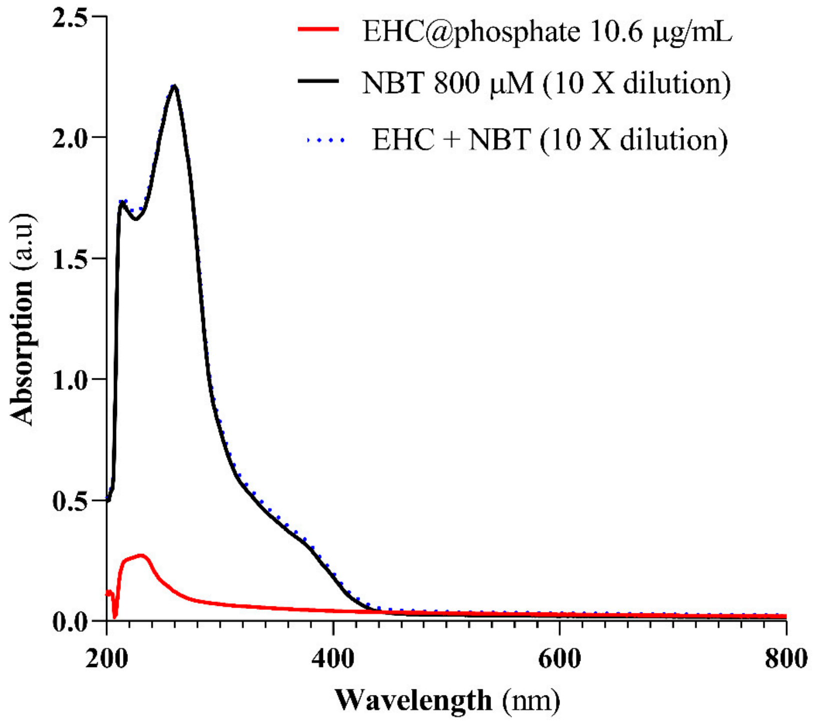

Figure S3. Spectra of EHC, NBT and EHC+NBT solutions. These spectra provide evidence that NBT is not adsorbed onto EHC. Otherwise, a decrease and/or wavelength shift on the NBT absorbance peak would be observed. Thus, the presence of the EHC nanomaterials does not diminish the NBT reduction capacity.

Table S1 - Conditions used for the SOD-like activity of EHC nanomaterials assay.

		**EHC concentration in the reaction medium***						
(μg/mL)		**0**	**2.1**	**4.3**	**6.4**	**8.5**	**10.6**	**13.2**
		**Volumes used in SOD assays**						
Buffer[1] (μL)		2050	1925	1800	1675	1550	1425	1214
EHC solution[2] (μL)		0	125	250	375	500	625	776
NBT[3] (μL)		200	200	200	200	200	200	200
XO[3](μL)		50	50	50	50	50	50	50
5 minutes incubation at 37 ºC								
HX[3] (μL)		200	200	200	200	200	200	200
Kinetics followed at 560 nm								
		**Corresponding Ionic Strength (M)**						
Buffer + NBT + XO + HX		0.395	0.3753	0.3555	0.3358	0.3160	0.2963	0.2724
EHC prepared in 0.05 M phosphate buffer (I = 0.1 M)		0	0.0050	0.0100	0.0150	0.0200	0.0250	0.0310
EHC prepared in 0.2 M phosphate buffer (I = 0.4 M)		0	0.0200	0.0400	0.0600	0.0800	0.1000	0.1242
Total ionic strength	minimum	0.3950	0.3803	0.3655	0.3508	0.336	0.3213	0.3034
	maximum	0.3950	0.3953	0.3955	0.3958	0.396	0.3963	0.3966

[1]0.1 M KH_2PO_4 + 5 mM Na_2EDTA (pH 7.40);

[2][EHC]= 42.53 ± 0.84 μg/mL (prepared in phosphate buffer);

[3][NBT] = 10 mM; [XO] = 0.4 mg/mL; [HX]i= 2.5 mM; prepared in 0.1 M KH_2PO_4 + 5 mM Na_2EDTA solution (pH 7.40);

*For each EHC concentration tested, a paired control test was also carried out, in which the nanomaterial solution was replaced by an equal volume of electrolyte solution with the same composition, pH, and ionic strength;

Table S2 - Conditions used for the SOD activity assay.

	**SOD concentration in the reaction medium**							
(μg/mL)	**0**	**0.18**	**0.92**	**1.84**	**3.68**	**7.36**	**14.72**	**18.40**
	**Volumes used in SOD assays**							
Buffer (μL)	2050	2045.4	2027	2004	1958	1866	1682	1590
SOD (μL)	0	4.6	23	46	92	184	368	460
NBT[1] (μL)	200	200	200	200	200	200	200	200
XO[1] (μL)	50	50	50	50	50	50	50	50
5 minutes incubation at 37 ºC								
HX[1] (μL)	200	200	200	200	200	200	200	200
Kinetics followed at 560 nm								
	**Corresponding Ionic Strength (M)**							
Buffer + NBT + XO + HX + SOD[2]	0.395							

[1][SOD]i =100 μg/mL; [NBT]i = 10 mM; [XO]i = 0.4 mg/mL; [HX]i = 2.5 mM;

[2]The ionic strength of the buffer is 0.395 M; NBT, XO, HX, and SOD were prepared in this buffer solution (0.1 M KH_2PO_4 + 5 mM Na_2EDTA, pH 7.40), therefore, in this case, the ionic strength remains the same throughout all assays;
